# Humoral immune response to *Plasmodium falciparum* vaccine candidate GMZ2 and its components in populations naturally exposed to seasonal malaria in Ethiopia

**DOI:** 10.1186/1475-2875-12-51

**Published:** 2013-02-05

**Authors:** Hassen Mamo, Meral Esen, Anthony Ajua, Michael Theisen, Benjamin Mordmüller, Beyene Petros

**Affiliations:** 1 Microbial, Cellular and Molecular Biology Department, College of Natural Sciences, Addis Ababa University, P. O. Box 1176, Addis Ababa, Ethiopia; 2 Institute of Tropical Medicine, University of Tübingen, Wilhelmstraße 27, Tübingen, 72074, Germany; 3 Centre de Recherche Médicale de Lambaréné (CERMEL), Lambaréné, BP 118, Gabon; 4 Department of Clinical Biochemistry and Immunology, Statens Serum Institut, Artillerivej 5, Copenhagen S 2300, Denmark; 5 Center for Medical Parasitology at Department of International Health, Immunology and Microbiology, University of Copenhagen, Bartholinsgade 2, Copenhagen K 1356, Denmark

**Keywords:** Plasmodium falciparum, Malaria, Ig, ELISA, Ethiopia, Vaccine, Antigen, Cytophilic, Noncytophilic

## Abstract

**Background:**

In Ethiopia, the general population is vulnerable to unpredictable epidemics of *Plasmodium falciparum* malaria. However, there is little information on the anti-malaria immune profile of the population in the endemic regions of the country.

**Methods:**

The study was designed to investigate the nature of humoral immune response to malaria in two ethnic groups in two endemic localities: Shewa Robit in north, and Boditi in south Ethiopia which are characterized by varying levels of malaria transmission and altitude. In a cross-sectional study, the study participants were diagnosed for malaria infection microscopically and by the rapid diagnostic test (RDT). Sera were tested by using enzyme-linked immunosorbent assay (ELISA) for total immunoglobulin (Ig) G against *P. falciparum* blood-stage vaccine candidate GMZ2 and its subunits (Glutamate-rich protein (GLURP-R0), merozoite surface protein 3 (MSP3); as well as IgG subclasses against GLURP-R0 and MSP3.

**Results:**

Whereas 23(8.6%) blood smear-positive cases for *P. falciparum* were detected in Boditi*,* all Shewa Robit study participants had no detectable *P. falciparum* infection. In both localities, total IgG prevalence and levels to GMZ2 were significantly higher than the response to the component domains indicating the strong recognition of GMZ2 by antibodies acquired through natural exposure. Total IgG and subclass prevalence and levels were higher in Shewa Robit than Boditi, suggesting difference in the intensity of malaria transmission in the two localities and/or genetic differences between the two populations in their response to the antigens. In both study sites, IgG subclass levels to GLURP-R0 were significantly higher than that to MSP3 for all corresponding subclasses in most individuals, indicating the higher relative antigenicity and probably protective potential of GLURP-R0 compared to MSP3. Against both GLURP-R0 and MSP3, the ratio of cytophilic to noncytophilic antibodies was >1 in the majority of the study participants, in both study sites, suggesting the induction of protective (cytophilic) antibodies against the two antigens. Analysis of age-related pattern in antibody levels against the antigens showed a positive association with increasing age.

**Conclusions:**

*P. falciparum* GLURP-R0 and MSP3 separately as well as in a fused form in GMZ2 are readily recognized by the sera of the study populations. The significantly higher antibody prevalence and level detected against GMZ2 compared to either of its subunits separately, in naturally exposed populations, suggests the synergistic effect of GLURP-R0 and MSP3 and that GMZ2 could be a more relevant blood-stage malaria vaccine candidate than the individual components. Detection of high-level antibody responses in non-febrile, smear-negative individuals may possibly be an indication of a low-grade, asymptomatic sub-microscopic infection in the induction and maintenance of high-level malaria immunity.

## Background

Globally, over 225 million malaria cases and 781,000 deaths occur annually [1]. *Plasmodium falciparum* is the most widely distributed and deadly species. Drug resistance of the parasite and insecticide resistance of the vector prove the fight against the disease is challenging. An effective malaria vaccine would be integrated into existing control strategies and make malaria elimination/eradication plans more feasible. Various malaria candidate vaccines are at different clinical trial levels; until now only one of them, the RTS,S vaccine candidate, completed Phase III [2]. A highly effective malaria vaccine is expected to be a combined multi-stage, multi-component and as such a blood-stage component must be included. But little is understood pertaining to the immune correlates of protection and the appropriate antigens that elicit the relevant immunological machinery. The concern increased when two blood-stage vaccines, falciparum malaria protein 1 representing the 42-kDa C-terminal fragment of merozoite surface protein 1 (MSP1) formulated with AS02 (FMP1/AS02) and apical membrane antigen 1 (AMA1)-C1/Alhydrogel, in Phase IIb trials failed to confer protection despite eliciting high antibody levels [3,4]. Individuals in endemic areas may develop high levels of antibodies, but these antibodies may not necessarily confer the desired protection. In view of this, it was hypothesized that the absence of protection may be because of an imbalance in immunoglobulin (Ig) G (IgG) subclass pattern [5]. Blood-stage parasites are mainly attacked by IgG1 and IgG3 cytophilic, whereas IgG2 and IgG4 noncytophilic may block the protective activity of cytophilic antibodies [6].

While MSP3 is an erythrocytic-stage protein glutamate-rich protein (GLURP) is expressed in both pre-erythrocytic and erythrocytic stages of *P. falciparum*[7]. A 220-kDa glutamate-rich protein (GLURP) has a relatively conserved N-terminal non-repeat region containing amino acids 27–500 (fragment R0), a central repeat region with amino acids 500–705 (fragment R1), and a C-terminal immunodominant repeat region of amino acids 705–1178 (fragment R2) [8]. A major B-cell epitope of the 48-kDa MSP3, which is conserved between different *P. falciparum* isolates, is the C-terminal region, representing amino acids 212–318 [9]. GMZ2 is a secreted fusion protein produced in *Lactococcus lactis* from genetically coupled *P. falciparum* GLURP-R0 and MSP3_212-380_[10]. Phase I clinical trials of the candidate vaccine conducted in malaria naïve [11] and naturally exposed individuals [12] demonstrated the immunogenicity, tolerability and safety of GMZ2. Anti-GLURP-R0 and -MSP3 antibody responses may have various forms of possible interactions – antagonistic, synergistic or no interaction under natural settings. The objective of this study was to produce baseline data on antibody responses to GMZ2 and its component domains in two epidemic-prone settings in Ethiopia.

## Methods

### Study sites

**Figure 1:**
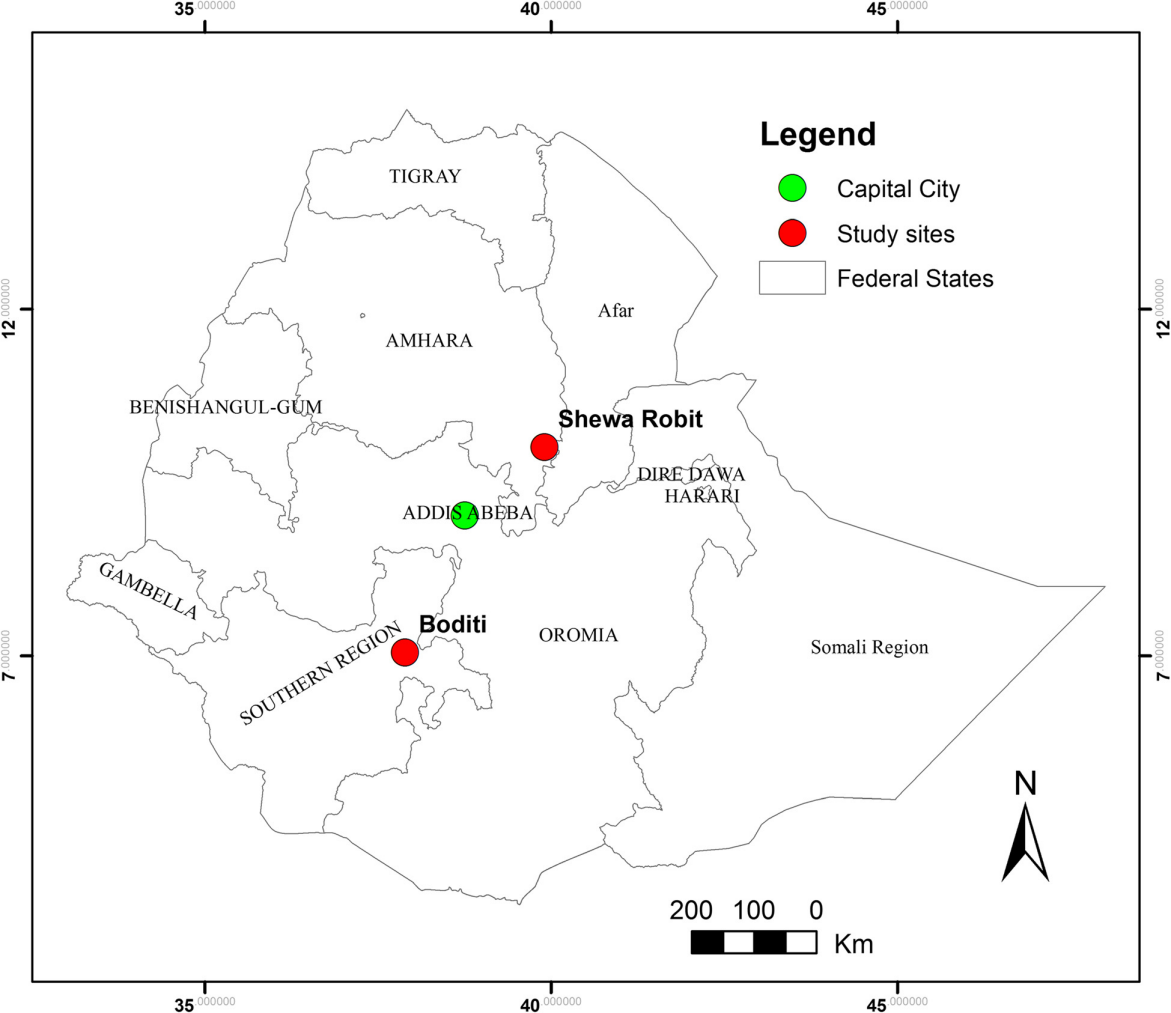
Map of Ethiopia showing the location of the two study sites, Boditi and Shewa Robit.

The study was carried out in Shewa Robit, a town in north-central Ethiopia located about 225 km north of Addis Abeba with geographic coordinates 10°0′36″ N latitude and 39°54′10.8″ E longitude and Boditi located in south western Ethiopia, about 370 km away from the capital having coordinates of 7°2′16.8″ N latitude and 37°53′16.8″ E longitude (Figure 1). Both areas receive high rainfall during the main rainy season (June to September) and are characterized by markedly unstable seasonal malaria. Most individuals in the study sites are expected to be exposed to falciparum malaria at least once in life time. Nationwide, *P. falciparum* and *P. vivax* are the dominant malaria parasites in Ethiopia accounting for about 60% and 40% of total malaria cases, respectively. *An. arabiensis* is main disease vector, while *An. pharoensis*, *An. funestus* and *An. nili* occasionally transmit the disease in certain areas of the country [13]. However, no data are currently available pertaining to malaria transmission dynamics, including annual entomological inoculation rates and prevalence estimates concerning the two study villages.

### Ethical considerations

The study was approved by the Ethics Review Committee of Department of Biology, Addis Abeba University and had a national ethical clearance from the Ethiopian Federal Ministry of Science and Technology. Informed consent was obtained from adult participants and parents/legal guardians for children prior to sampling. Malaria smear-positives were treated free of charge as per the national treatment guideline (*P. falciparum*: artemether–lumefantrine, *P. vivax*: chloroquine).

### Study design, sampling and infection detection

**Figure 2:**
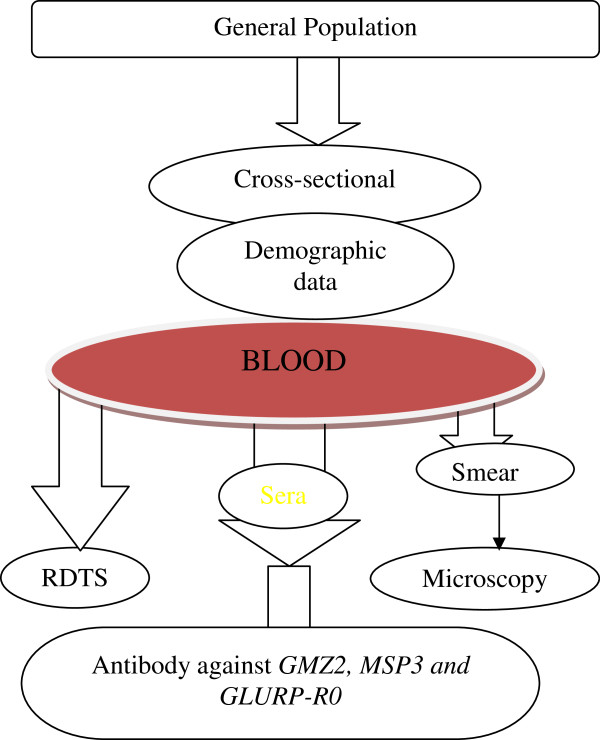
**Study design of antibody analysis against *P. falciparum *blood-stage vaccine candidate antigens in Boditi and Shewa Robit.**

Cross-sectional surveys took place in 2008 (Shewa Robit) and 2009 (Boditi), both in the dry season when there was minimum malaria transmission. Five ml of venous blood was collected from each participant. Malaria RDT using CareStart™ malaria HRP2/pLDH(Pf/pan) Combo Cassette Test (Access Bio, Inc) was carried out as per the manufacturer’s instruction. Thick and thin blood smears were prepared and microscopically screened for *Plasmodium* infection. Sera were separated and stored at -20°C until use for immunological assessment. The flow chart of the study design is shown in Figure 2.

### Elisa

Total IgG was measured against GMZ2, GLURP-R0 and MSP3, and IgG subclass levels for GLURP-R0 and MSP3. All ELISA experiments were performed following a previously published test protocol [11]. In brief, microtiter plates (NUNC Maxisorp™, Germany) were coated with GMZ2 or GLURP at a concentration of 0.5μg/ml and for MSP3 1μg/ml diluted in phosphate buffered saline (PBS). The coated plates were washed four times after overnight incubation at 4°C with a washing buffer (PBS, 0.1% Tween (T)-20, 0.5M NaCl). The plates were blocked using blocking buffer (PBS, 3% non-fat milk powder, 0.1% T-20) and incubated in a room temperature for 1 h. Optimum dilutions (1:400 for total IgG and 1:100 for IgG subclasses) in dilution buffer (PBS, 1% non-fat milk, 0.1% T-20) were selected prior to the test procedure. Sera were loaded and the plates were incubated for 2 h in a room temperature on a shaker. After washing horseradish peroxidase (HRP)-conjugated goat anti-human IgG (Caltag, USA), diluted 1:3000 in dilution buffer, was added and incubated for 1 h to detect malaria-specific IgG. After washing 3,3',5,5'-tetramethylbenzidine (TMB-ONE) (KEM EN TEC, Belgium) was loaded as a substrate for the HRP and incubated in dark and the reaction stopped by 0.2M H2SO4 (32%, Merck, Germany) after 20 min. Optical density (OD) was determined at 450 nm (reference 620 nm) by plate reader (Asys Expert 96, Type G018065). For IgG subclass analysis one additional step of incubation was carried out with HRP-conjugated goat anti-mouse IgG (1:3,000) (Invitrogen, UK) after capturing the subclasses with mouse anti-human monoclonal IgG1 (1:3,000) or IgG3 (Skybio, UK), IgG2 (1:5,000) (Sigma, Germany) and IgG4 (1:3,000) (Caltag, USA). Positive reference serum from Gabonese donors (Lambaréné) and negative control sera from malaria naïve German donors were utilized for assay standardization. The cut-off threshold was determined as the mean plus three standard deviations (SD) from negative-control sera.

### Data analysis

The data was double entered into separate Microsoft Excel spreadsheets and checked for overall correctness and normality. As mean antibody levels were not normally distributed, nonparametric tests were used for analyses. Fisher’s exact test was used to test the significance between differences in antibody positivity rates for the antigens within and between the two study sites as well as between age groups, slide-positives and –negatives and between IgG subclasses. To test the difference in OD levels between more than two groups the Kruskal-Wallis test was used. Differences between median OD values for each antigen between two groups (pairwise) were analyzed by Mann–Whitney U (Wilcoxon rank-sum) test. Multiple comparisons were used to assess between which particular age groups (for more than two age groups) the significant differences existed. Spearman rank correlation was used to test the correlation between antibody level to each antigen and the age-related pattern of antibody level. Age-stratified analysis was followed for comparisons between the two study sites. Analysis was done using SPSS version 17.0 for Windows (SPSS Inc, Chicago, IL, USA). Two-sided *p*<0.05, 95% confidence intervals (CI) were considered significant.

## Results

### Study participants

Some 228 serum samples were collected from participants in Shewa Robit. The mean age was 28 years and there were no individuals below five years of age (range 5–80 years). All participants were asymptomatic and had no microscopically or RDT detectable *Plasmodium* infection at the time of sampling. A total of 265 serum samples was collected from residents of Boditi with mean age of 23 years. The highest age was 80 and the lowest was one year. Table 1 shows demographic characteristics of the studied populations. Some 44 individuals were febrile (axillary temperature ≥37.5°C). Eleven (25%) among febrile group were smear-positive for *P. falciparum*. Twelve asymptomatic infections were detected making the total number of positives 23(8.6%). Only one *P. vivax* infection was detected by microscope.

**Table 1:** Demographic characteristics of study participants from Shewa Robit and Boditi in Ethiopia

**Study site**	**Age groups**	**Male (n, %)**	**Female (n, %)**	**Total (n, %)**
Shewa Robit (N= 228)	5 -14	7 (3.0)	13 (5.7)	20 (8.7)
	> 14	181 (79.4)	27 (11.8)	208 (91.2)
Boditi (N = 265)	0 - 4	22 (8.3)	22 (8.3)	44 (16.6)
	4.1 - 14	51 (19.2)	36(13.6)	87 (32.8)
	>14	40 (15.1)	94 (35.5)	134 (50.6)

n = number of volunteers, % = percentage.

### Antibody prevalence and level

Antibody levels to GMZ2, GLURP-R0 and MSP3 were at detectable level in most individuals in both sites (Table 2). Positivity rates and levels to GMZ2 were significantly higher than that to MSP3 and GLURP-R0 (*p* < 0.001). Anti-GLURP-R0 total IgG positivity rate was higher than those to MSP3 in Boditi and the reverse was observed in Shewa Robit but the differences were not significant in both cases. Total IgG positivity rates were higher in Shewa Robit than Boditi for all the three antigens though all the differences were not significant. Total IgG levels to GMZ2 were significantly higher than that to MSP3 or GLURP-R0 in both sites (*p* <0.001). Total IgG levels to GLURP-R0 were higher than that to MSP3 but significant difference was observed only for Shewa Robit (*p* = 0.012). For all the three antigens tested, total IgG positivity rates were higher in Shewa Robit than Boditi though the differences were not significant. Total IgG levels were significantly higher for GMZ2 (*p* = 0.013) and GLURP-R0 (*p* = 0.021) in Shewa Robit than Boditi between age-stratified groups.

**Table 2:** **Positivity and levels of total IgG to *P. falciparum *GMZ2 and its component vaccine candidate antigens in sera from Boditi and Shewa Robit in Ethiopia**

	**Positivity rate (n, %)**		**Level**	
			**Median OD (interquartile range (IQR)**	
	**Boditi**	**Shewa Robit**	**Boditi**	**Shewa Robit**
	**(N=265)**	**(N=228)**	**(N=265)**	**(N=228)**
GMZ2	240 (90.6)	226 (99.6)	0.416(0.36-0.49)	0.540(0.46-0.62)
GLURP-R0	226 (85.6)	206 (90.4)	0.284(0.22-0.33)	0.366(0.28-0.48)
MSP3	216 (81.5)	215 (94.3)	0.264(0.21-0.29)	0.272(0.23-0.36)

There were some inter-individual variations in IgG reactivity against GLURP-R0 and MSP3 antigens in both study sites. An individual who showed high response for one antigen did not necessarily have a high response for the other. In Boditi, while a high proportion (71.3%) of serum samples were IgG positive against GMZ2, GLURP-R0 and MSP3 only 6.8% did not have a detectable IgG level against any of the antigens.

### Antibody responses by age and smear-positivity

**Figure 3:**
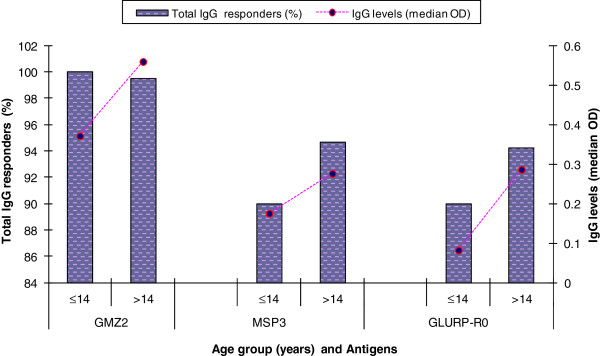
**The positivity and levels of total IgG antibodies to *P. falciparum, *GLURP-R0, MSP3 and GMZ2 in subjects of different ages from Shewa Robit.** The bars represent the proportion of total IgG responders and the broken line with filled circles represent levels (median OD) of the response.

For Shewa Robit, the age group over 14 years old had greater number of positive individuals than those under 14 for GLURP-R0 and MSP3 (Figure 3) though a significant difference was not found for all the three antigens. But the median total IgG levels to the antigens were significantly higher for the older age group for all the three antigens (*p <* 0.01) (Figure 3).

**Figure 4:**
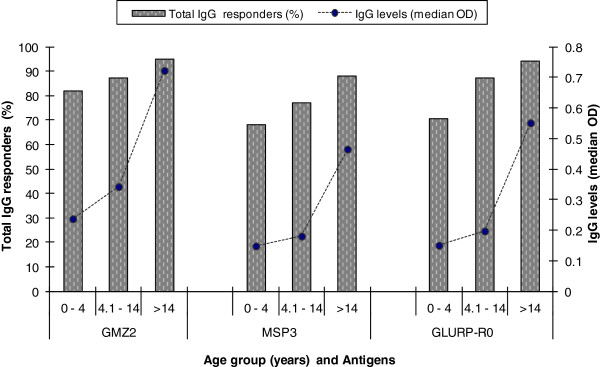
**The positivity and levels of total IgG antibodies to *P. falciparum, *GLURP-R0, MSP3 and GMZ2 in subjects of different ages from Boditi.** The bars represent the proportion of total IgG responders and the broken line with filled circles represent levels (median OD) of the response.

For Boditi, there was age-related increase in total IgG prevalence (Figure 4). There was a significant difference between age groups under four years and those above 14 in IgG positivity to GMZ2 (*p* = 0.005), MSP3 (*p* = 0.015) and GLURP-R0 (*p* <0.001). Levels of total IgG also showed an age-dependent increase with significant differences between all the three age groups for GMZ2, between under-four years and above 14, and between 5–14 years and above 14 for MSP3 and GLURP-R0 (*p* <0.001) (Figure 4). Though not significant, the prevalence and levels of total IgG to the different antigens were higher in the smear-negative group compared to smear-positives for *P. falciparum* (Table 3).

**Table 3:** **The positivity and levels of total IgG antibodies against MSP3, GLURP-R0 and GMZ2 in individuals with different *P. falciparum *parasitaemia status at the time of sampling in Boditi**

**Antigens**							
**Parasitaemia status**	**n**	***GMZ2***		***MSP3***		***GLURP-R0***	
		**Positivity**	**Level**	**Positivity**	**Level**	**Positivity**	**Level**
		**No. (%)**	**(Median OD(IQR)**	**No. (%)**	**(Median OD(IQR)**	**No. (%)**	**(Median OD(IQR)**
Negative	242	210 (86.7)	0.435(0.35	200 (82.6)	0.216	208	0.272
			0.49)		(0.17-0.24)	(85.9)	(0.21-0.31)
Positive	23	18 (78.2)	0.236(0.19-0.242)	16 (69.5)	0.164 (0.14-0.17)	18	0.186
						(78.2)	(0.15-0.21)

### IgG subclass distribution

Positivity rates of IgG1 and IgG3 to GLURP-R0 and all subclasses to MSP3 were significantly higher in Shewa Robit than Boditi between age-stratified groups (Table 4). Similarly, IgG1 (*p* = 0.023) and IgG2 (*p* < 0.001) antibody levels to MSP3 were significantly higher in Shewa Robit than Boditi between age-stratified groups. More samples were positive to GLURP-R0 but not MSP3 for all subclass antibodies in both sites. Also, antibody levels were significantly higher for GLURP-R0 than MSP3 for IgG1, IgG2 and IgG3 (*p* < 0.001) in both study populations. Although it may not necessarily reflect the real antibody concentration level, in terms of OD values in the majority of the study populations cytophilic subclasses were dominant over the noncytophilic ones for both antigens in both study sites.

**Table 4:** **The positivity and levels of IgG subclasses to *P. falciparum *GLURP-R0 and MSP3 in the sera of residents from Boditi and Shewa Robit**

**Antibody**	***GLURP-R0***				***MSP3***			
	**Positivity rate**		**Level**		**Positivity rate**		**Level**	
	**(n, %)**		**(Median OD (IQR)**		**(n, %)**		**(Median OD (IQR)**	
	**Boditi**	**Shewa Robit**	**Boditi**	**Shewa Robit**	**Boditi**	**Shewa Robit**	**Boditi**	**Shewa Robit**
IgG1	171	192	0.302	0.337	146	209	0.140	0.230
	(64.5)	(84.2)	(0.26-0.32)	(0.28-0.38)	(55.1)	(91.7)	(0.13-0.18)	(0.19-0.25)
IgG2	203	178	0.246	0.324	89	213	0.055	0.279
	(76.6)	(78.1)	(0.21 0.28)	(0.25-0.36)	(33.6)	(93.4)	(0.03-0.09)	(0.21-0.31)
IgG3	147	171	0.135	0.197	108	146	0.096	0.107
	(55.5)	(75.0)	(0.15-0.14)	(0.16-0.21)	(40.8)	(64.0)	(0.04-0.12)	(0.09-0.12)
IgG4	73	72	0.018	0.067	32	74	0.026	0.088
	(27.5)	(31.5)	(0.014-0.019)	(0.01-0.09)	(12.1)	(32.4)	(0.0.01-0.04)	(0.05-0.11)

## Discussion

The significantly higher total IgG prevalence and level detected in both study sites of the present study for GMZ2 than either of its two component domains indicates the enhanced antigenicity of GMZ2. The findings suggest that people in endemic areas have high antibody levels to the GLURP and MSP3 components of GMZ2 antigen, which is part of the GMZ2 vaccine candidate, in the absence of microscopically detectable infection. The higher total IgG and subclass levels against GLURP-R0 than MSP3 in both study sites indicates the better antigenicity of GLURP-R0 compared to MSP3. The findings are in line with data from pre-clinical and clinical evaluations of the GMZ2 vaccine candidate. Pre-clinical studies in mice [10] and monkeys [14] documented higher total IgG prevalence and level against GMZ2 compared to GLURP-R0 and MSP3. The same studies showed that total IgG prevalence and level were significantly higher for GLURP-R0 than MSP3.

Initial clinical trials in malaria-naïve [11] and naturally exposed individuals [12] to assess safety and immunogenicity of GMZ2 have shown stronger anti-GMZ2 responses than the responses to its component antigens, with GLURP-R0 being relatively more immunogenic than MSP3. Studies that examined anti-GLURP-R0 and -MSP3 natural responses separately had recorded varying total IgG prevalence for the two antigens. In one such report, anti-MSP3 total IgG prevalence was higher than that for GLURP-R0 in a hyper-endemic area in Myanmar [15]. Also, a study from Venezuelan Amazon showed higher total IgG prevalence against GLURP-R0 than MSP3 [16].

The inter-individual variability in antibody response to a specific antigen observed among the study participants was also reported from other malaria endemic areas [17]. In the present study an individual who showed high response for MSP3 did not have necessarily high response for GLURP-R0 suggesting that no predisposing host factors are responsible for induction of high antibodies; it may be due to different antigen characteristics.

Currently, knowledge on the durability of antibody responses to *P. falciparum* vaccine antigens and how they are maintained remains poorly defined. In this study, in the context of heavily reduced transmission, strong immune responses were recorded for all of the antigens in most study participants. This suggests the stability of malaria immunity. The data provided additional evidence in support of earlier observations that antibodies can last for years in the absence of significant boost infections [18,19].

After one year, antibody level to GMZ2 remained high in the first clinical trial in malaria-naïve adults although it was lower compared to the level on day 84 [11]. In the absence of boost infection, anti-GMZ2 antibodies in immunized adults and children lasted six to 12 months [12] and then started waning. When natural MSP3- and GLURP-R0-specific IgG subclass levels were considered, no major change in the levels of specific IgG1 against the two antigens before and after five years was reported [15]. The report however indicated that the protection status of some individuals was associated with IgG3 to GLURP-R0 at one time point and IgG3 to MSP3 after five years suggesting changes between individuals in their response to the antigens and the dynamics (or complementing effect) of responses to these antigens over time.

The present study suggests that both antigens, GLURP-R0 and MSP3, are important for the induction of protective antibodies against malaria from natural infections providing additional rational for combining the two antigens in a hybrid vaccine formulation. Thus GMZ2 has high potential as a candidate vaccine compared to either GLURP-R0 or MSP3 candidate vaccine antigens. However, it remains unclear whether the GMZ2 vaccines would be effective in the heterogeneous epidemiological settings of Ethiopia as there is evidence that vaccine-induced antibodies may not be as potent as those naturally acquired [20]. But the induction of functional antibodies, cytophilic subclasses, following natural exposure signals that the GMZ2 vaccine would likely be effective at least through boosting the pre-existing antibodies. The role of challenge infections in boosting vaccine stimulated-antibodies for parasitaemia control was noticed in pre-clinical study in monkeys [14]. Furthermore, there is evidence from Phase I clinical trial in naturally exposed adults that the GMZ2 vaccine boosted pre-existing natural immunity [12]. In some studies anti-GLURP antibodies were associated with protection [21], in others not [22]. The association of antibody levels to MSP3 with protection has also been described [23].

The overall dominance of cytophilic IgG subclasses over the noncytophilic shows the functionality of GLURP-R0-specific IgG subclasses explaining the ‘better protected’ status of Shewa Robit participants who were asymptomatic and microscopy negative. The dominance of MSP3-specific IgG1 and IgG3 isotypes in Boditi sera goes in agreement with studies from other settings [21,24]. In both study sites, IgG subclass antibody level to GLURP-R0 was significantly higher than that to MSP3 for all corresponding subclasses characterized in most individuals indicating the increased relative antigenicity and protective potential of GLURP-R0 compared to MSP3.

Factors that control the preferential induction of polarized subclass responses are not well understood *in vivo*. Cytokines and B cell activators are involved in inducing Ig class switching in a model system [25]. It was suggested that some bacterial antigens are known to induce IgG2 expression [26] and allergens or helminths stimulate IgG4 and IgE induction [27]. Further, polysaccharides such as dextran or levan are known to contribute towards IgG2 elevation [28] especially in response to other parasitic diseases like schistosomiasis [29]. Malaria-specific subclass pattern and relative proportions may differ from population to population depending on various factors such as level of malaria transmission, human genetics and age, and type of antigen epitope involved [30].

The age-related increase of antibody levels observed for the antigens in Shewa Robit is in agreement with other studies [22,31,32]. Although not significant, antibody levels to the antigens in the study were higher in smear-negative group for *P. falciparum* infection in Boditi sera. This suggests that smear-negative individuals were relatively protected due to their high antibody levels. The prevalence of IgG to MSP2 serogroup antigens in a seasonal transmission area correlated positively with presence of parasitaemia at the time of sampling [33]. Such reports may suggest the influence of recent boost infections that resulted in relatively elevated antibody responses in one hand and the lower protective efficacy of the antibodies in parasite control on the other. Nonetheless, in another study, no relationship was observed between antibody responses to GLURP-R0 and GLURP-R2 and presence or absence of *P. falciparum* in blood and the level of parasitaemia [34].

The significantly lower falciparum-specific antibody level noticed in Boditi than Shewa Robit may be due to genetic differences between the two population groups, parasite strain variation in the localities, or level of malaria transmission. Boditi is a highland fringe at an altitude of 2,059 m above sea level (masl). The seasonality of malaria in highlands is more pronounced with characteristic lower immunity compared to warm hotbeds. A classical serological survey conducted in Ethiopian highlands indicated that high *P. falciparum*-specific responses were obtained for people living at elevations of 1,828 masl and less compared to those at 1,920 or above [35]. Treatment-seeking behaviour and access to prompt effective treatment schemes, possession and appropriate usage of insecticide-treated bed net (ITN), vicinity to mosquito breeding sites, regularity in indoor residual spray (IRS); and the overall variations of control methods may account for heterogeneity in exposure to infective mosquito bites, making the level of boost infections more infrequent thereby possibly contributing towards relatively lower antibody levels in Boditi study participants. In Shewa Robit (at an elevation of 1,380 masl) an irrigated tobacco farmland created ideal pools of mosquito breeding sites. This can be a potential source of submicroscopic, subpatent infections that maintained higher immunity compared to Boditi. The Shewa Robit study participants who had stronger functional antibodies were asymptomatic and had no microscopically detectable infection suggesting that they were relatively better protected compared to the Boditi.

## Conclusions

Detection of high antibody prevalence and level in smear-negative individuals suggests that people in seasonal malaria transmission areas of Ethiopia acquire and maintain immune responses to *P. falciparum* blood-stage antigens in the absence of microscopically detectable infection. Higher antibody prevalence and level detected against GMZ2, compared to its subunits individually, suggests that this antigen could be a more relevant blood-stage malaria vaccine candidate. Though a few sera did not show reactivity to GLURP-R0 and MSP3 in both study sites, most were positive for all IgG1, IgG2 and IgG3 subclasses suggesting that the antigens are readily recognized in the populations. The ratio of cytophilic to noncytophilic antibodies against GMZ2 subunits was >1 in the majority of the study population in both study sites implying the stimulation of functional IgG subclasses. The highest antibody prevalence and level were among adults and varied between antigens.

## Abbreviations

ELISA: Enzyme-linked immunosorbent assay; AMA: apical membrane antigen; FMP: Falciparum malaria protein; GLURP: Glutamate-rich protein; Ig: Immunoglobulin; IRS: Indoor residual spray; IQR: Interquartile range; ITN: Insecticide-treated bed net; MSP: Merozoite surface protein; TMB: Tetramethylbenzidine; PBS: Phosphate buffered saline; HRP: Horseradish peroxidase; OD: Optical density; CI: Confidence interval; SD: Standard deviation; UK: United Kingdom; USA: United States of America.

## Competing interests

The authors declare that they have no competing interests.

## Authors’ contributions

HM contributed towards the conception and design of the study; acquisition, statistical analysis and interpretation of the data; and drafting and revision of the manuscript. ME supervised laboratory work, interpreted the data and revised the manuscript. AA participated in laboratory investigations, interpreted the data and revised the manuscript. MT interpreted the data and revised the manuscript. BM supervised laboratory work, interpreted the data and revised the manuscript; BP conceived the study, participated in its design, supervised fieldwork, interpreted the data and revised the manuscript. All authors read and approved the submission of the manuscript for publication.
